# Etiologies of non-genetic epilepsies of child and adolescent, newly diagnosed in Ouagadougou, Burkina Faso

**DOI:** 10.11604/pamj.2018.31.175.17074

**Published:** 2018-11-13

**Authors:** Djingri Labodi Lompo, Ousséini Diallo, Ben Aziz Dao, Romaric Bassole, Christian Napon, Jean Kabore

**Affiliations:** 1 University Hospital of Tingandogo, Unit of Formation and Research of the Sciences of the Health, University Ouaga I-Pr Joseph Ki-Zerbo, Ouagadougou, Burkina Faso; 2 University Hospital of Yalgado Ouedraogo of Ouagadougou, Unit of Formation and Research of the Sciences of the Health, University Ouaga I-Pr Joseph Ki-Zerbo Ouagadougou, Burkina Faso

**Keywords:** Epilepsy, non genetic, brain scan, etiologies

## Abstract

**Introduction:**

in sub-Saharan Africa, epilepsy is common and mainly concerns children before the age of 15 years. The data on childhood epilepsy is parcel, but a high prevalence of non-genetic epilepsy is frequently reported. EEG, TDM and MRI devices are rare. The aim of this work was to study the etiological aspects of non - genetic epilepsy of the child and adolescent, newly diagnosed in Ouagadougou, Burkina Faso.

**Methods:**

This was a cross-sectional, descriptive, multicentric study, from 01/01/2016 to 31/12/2016, involving patients aged 0 to 18 years old, epileptic, newly diagnosed, in the city of Ouagadougou. Each patient included in the study was to have had an EEG and brain CT scan and/or brain MRI and to gather the anamnestic and electro clinical arguments for non-genetic epilepsy. Sociodemographic, clinical, EEG and neuroradiological data were analyzed. An univariate analysis was used to determine the electro-clinical and neuro-radiological characteristics associated with epilepsies of structural causes.

**Results:**

In all 115 patients were collected, with an average age of onset of epilepsy of 8.2 years, a male predominance with a sex ratio to 1.67. Risk factors of epilepsy was present in 74.8%; They were dominated by perinatal events in 79.1%. Focal seizures, daily frequency of these seizures and focal epilepsy, were predominant, respectively in 53%, 58% and 60.9% of cases. Brain scan and Brain MRI where performed in 90.4% and 9.6% of patients, respectively. The brain sequelaes of perinatal adverse events, the sequelae of central nervous system infections, and the sequelae of cranial and brain trauma, with 34.8%, 14.8%, and 5.2% respectively, were the main causes of non- genetic epilepsies of the child and adolescent. No cause was identified in 37.4% of cases.

**Conclusion:**

The improvement of policies in the field of maternal and child health and the generalization of the control of infectious and parasitic diseases, including malaria, may contribute to the reduction of non-genetic epilepsy in sub-Saharan Africa.

## Introduction

Epilepsy is a public health problem in developing countries, particularly in Burkina Faso, where prevalence would reach 5 to 10 times that of industrialized countries [1]. It mainly concerns children before the age of 15 years, although in sub-Saharan Africa (SSA) the available pediatric data are fragmentary [2, 3]. In SSA, the causal factors of non-genetic epilepsy of the child appear to be dominated by perinatal complications and central nervous system (CNS) infections, due to the combination of several factors, such as socio-cultural burdens, the high prevalence of unassisted home births, the existence of a endemic-epidemic infectious pathologies, the lack of adequate sanitary infrastructures and qualified human resources [4]. The risk of developing epilepsy in the cohorts of survivors of CNS infections is estimated at 26% in developing countries, particularly in SSA [5], compared with 6.8% in developed countries [6]. In SSA, CT scan and particulary MRI, are little available or not accessible, whereas they are indispensable for the identification of epileptogenic brain lesions and the causal diagnosis of certainty of certain epilepsy, including epilepsy of structural causes. In the absence of these neuro-imaging examinations in most of the studies in SSA, the certainty of the causative diagnosis of epilepsy is difficult to establish, because usually based on the history of the disease, which is not always recorded and therefore subject to a recall bias [1, 7]. The aim of our work was to study the causal aspects of non-genetic epilepsy of the child and adolescent, in Ouagadougou, Burkina Faso, according to electro clinical and neuroradiological diagnostic criteria.

## Methods

This was a cross-sectional, descriptive, multicentric study, which took place from 01 January 2016 to 31 December 2016. It concerned all children and adolescents, aged between 0 and 18 years of age, with newly diagnosed epilepsy, followed by an external neurology consultation in the various University hospitals of the city of Ouagadougou, Yalgado Ouedraogo, Tingandogo, pediatric Charles de Gaulle, and Medical Center with surgical antenna of Schiphra. To be included in the study, each epileptic patient should benefit from at least one EEG and brain imaging (brain CT scan and/or MRI) and bring together the anamnestic and electro clinical arguments for non-genetic epilepsy. Were not included in the study, patients who had seizures whose epileptic nature was not proven, those who had genetic epilepsy on the basis of the anamnestic, clinical and EEG arguments, those for whom the EEG and the neuro Imaging were not available and finally those at which consent could not be obtained. Patients were collected consecutively in external neurology or hospitalization, by senior neurologists, at the various collection sites. The data were collected from the interrogation, the clinical examination, data from the standard EEG, which was assembled in accordance with the international system 10/20 and data from the brain CT and/or MRI. The study variables concerned sociodemographic characteristics (age, sex, level of schooling), clinical characteristics (risk factors for epilepsy, age of onset of seizures, duration of crisis progression, frequency of seizures, psychomotor development, inter-critical neurological examination, classification of epileptic seizures and epilepsy performed retrospectively according to the classification of ILAE 2017), results of EEG and neuro Imaging (brain CT or Brain MRI). The data were analyzed using the CDC's EPI-Info software version 7.1.3.3. Descriptive statistics were used to summarize the data. The associations between the variables were tested by hypothesis testing (CHI2 test and Fischer test). The selected significance threshold was set at 5%. An univariate analysis was used to assess the associations between clinical, EEG and neuroradiological characteristics and epilepsy of structural causes. Ethical considerations: the study obtained the approval of the National bio-ethics Committee of Burkina Faso and was then carried out after authorisation by the administrations of the various hospitals, and after obtaining the informed consent of Parents of the different patients.

**Operational definitions:** Diagnostic criteria for genetic epilepsy: early childhood between the age of 4-10 years; possibility of genetic epilepsy in family history but absence of acquired risk factors for epilepsy in personal history; normal neurological, neuroradiological and neuropsychological assessment; rare and brief seizures often appearing in sleep; several possible forms of seizures (generalized seizures, generalized myoclonic seizures, typical absences); revealed to the EEG of paroxysmal inter-critical anomalies appearing on a normal background activity, increasing in sleep, most often favorable evolution with healing towards puberty. Non-genetic epilepsy: epilepsy that does not have the characteristics of genetic epilepsy, associated with the identification of brain, fixed or evolutionary structural lesions, of épileptogenicity already demonstrated, with positive anatomo-electro-clinical correlation; or epilepsy that does not have the characteristics of genetic epilepsy, whose associated electro-clinical characteristics are suspected of a causal structural anomaly, not yet identified by brain CT and/or brain MRI. Clinical, EEG and neuroradiological criteria, required for the diagnosis of non-genetic epilepsy of structural cause: clinical criteria: at least one of the following clinical characteristics: lack of arguments for a epileptic syndrome of genetic cause; presence of acquired epilepsy risk factors, except for genetics, in the history of patients; early onset of epilepsy; semiology can include all possible types of seizures; coexistence of focal neurological signs, cognitive deterioration, behavioral disorders and sometimes extra neurological signs suggesting an underlying disease responsible for epilepsy; EEG criteria: at least one inter critical EEG patterns, suggestive of underlying brain damage: abnormal, slow or asymmetric background activity; continuous slow wave, focused, associated or not to spikes, spikes waves or polypointes waves; independent multifocal paroxysmal anomalies; Diffuse paroxysmal anomalies with anterior predominance, symmetrical or predominant slow-wave spikes on one side, performing the appearance of a secondary bilateral synchrony; reduction or disappearance of focal inter critical abnormalities before the onset of the first clinical signs; polymorphic critical discharges; slow rhythmic waves and/or critical discharges without clinical translation; mandatory neuroradiological criteria: demonstrated to brain CT and/or brain MRI of fixed or evolutionary structural lesions of already demonstrated epileptogenicity, with positive anatomo-electro-clinical correlation. Epilepsy of unknown cause: epilepsy that does not meet the criteria of genetic epilepsy but for which the neuro imaging did not highlight structural brain lesions, fixed or evolutionary, with epileptogenicity already demonstrated, with positive electro-clinical correlation. It could be of radiological infra structural brain damage or metabolic, immune or infectious abnormalities not identified, because not explored in this study, because of the non availability or inaccessibility of their diagnostic methods in our work. Epilepsies associated with a history of perinatal events or infectious childhood history, reported to the interrogation but without brain injury of epileptogenicity demonstrated to imaging were considered as unknown cause. Classification of epilepsies and epileptic seizures has been made to the ILAE 2017.

## Results

We consecutively collected 115 patients with the characteristics of a non genetic epilepsy. The average age of patients and beginning of the seizures was 9.1 years (6 months and 18 years) and 8.2 years (28 days-18) respectively. The majority of patients were masculine with 72 cases (62.6%) with a sex ratio H/F of 1.67, were at school with 67 cases (58.3%), had risk factors for epilepsy in their history, 86 cases (74.8%); the risk factors of epilepsy were dominated history of perinatal events with 68 cases (59.1%) and history of acute meningo encephalitis of childhood with 13 cases (11.3%). Focal seizures with 61 cases (53%) were the most frequently encountered; the daily seizures, rated frequency in 67 patients (58%), were the most represented. Psychomotor disorders with 46 cases (40%), focal motor deficit, cerebral palsy with 16 cases (13.9%), hyperactivity and apathy with 12 cases each (10.4%), were the most frequently encountered neurological and neuropsychological anomalies. The epileptic paroxysms have been identified in 86 patients (74%); after electro clinical classification, focal epilepsies, generalised epilepsies and epilepsie with focal and generalized seizures, were respectively found in 70 cases (60.9%), 22 cases (19.1%) and 13 cases (11.3%). Table 1 summarizes the sociodemographic, clinical and EEG characteristics of patients. Brain CT scan and MRI were conducted respectively in 104 patients (90.4%) and 11 patients (9.6%). Neuroradiological anomalies have been identified in 72 patients (62.6%). The cortico-sub-cortical atrophy isolated or associated with other anomalies with 55 cases (47.8%), the limited hypodensities with 18 cases (15.6%) and porencephalic cavities with 12 patients (10.3%), were the more frequently encountered neuroradiological anomalies. Structural causes of epilepsy and unknown causes of epilepsy were diagnosed in 72 patients (62.6%) and 43 patients (37.4%), respectively. The structural causes were dominated by the sequelae of antenatal or perinatal cerebral suffering with 40 cases (34.8%), the sequelae of CNS infections with 17 cases (14.8%) and the sequelae of cranial and brain trauma with 6 cases (5.2%). Table 2 summarizes the neuroradiological characteristics and etiology of epilepsy of structural causes Structural causes were significantly associated with the groupe age of 28days to12 years (70.5%; p = 0,043), with prior epilepsy risk factors (75%; p < 0.0001), with starting age ranging from birth to age 12 (66.7 %; p = 0,013), with frequent seizures (73.8%; p = 0,0002), neurological clinical anomalies (90.7%; p < 0.0001), with psychomotor disorders (93.5%; p < 0.0001), with behavioural disorders (92.6%; p < 0.0001), with cerebral atrophy (72%; p < 0.0001) and porencephalic cavities (85%; p = 0.002).

**Table 1:** Sociodemographic, Clinical and EEG characteristics of patients

Characteristics	Numbers	Frequencies
School status		
Still in school	67	58.3%
Non schooled	41	35.6%
School eviction	7	6.1%
Epilpepsy risk factors	86	74.8%
Perinatal events	68	59.1%
*Embryofoetopthies*	5	4.3%
*Prematurity*	1	0.9%
*Acute neonatal suffering*	56	48.7%
*Acute neonatal infections*	3	2.6%
*Complicated febrile seizures*	3	2.6%
Post natal acute méningo-encephalitis	13	11.3%
Cranial and brain trauma	4	3.5%
Metabolic/toxic encéphalopathy	1	0.9%
Type of seizures		
Focal seizures	61	53%
Generalized seisures	31	27%
Non-classifiable seizures	23	20%
Frequencie of seizures		
Daily	67	58%
Weekly	17	15%
Monthly	30	26%
Rare seizures	1	1%
Intercritical clinical review		
Neurological examination anomalies	54	47%
Motor deficit	33	28.7%
Cerebral palsy	16	13.9%
Visual field deficit	6	5.2%
Sensory deficit	5	4.3%
Ataxia	3	2.6%
Psychomotor disorders	46	40%
Behavioural disorders	28	24.3%
Hyperactivity	12	10.4%
Apathy	12	10.4%
Frontal syndrome	4	3.5%
Depressive syndrome	2	1.7%
Autistic syndrome	2	1.7%
Bullying	1	0.9%
Epileptic paroxysmal anomalies at EEG	86	74%
Classification of epilepsy		
Focal epilepsies	70	60.9%
*Frontal*	33	28.7%
*Central*	15	13%
*Temporale*	14	12.2%
*Occipital*	8	6.9%
Generalized epilepsies	22	19.1%
Epilepsies combining generalized and focal seizures	13	11.3%
Epilepsies of indeterminate location	10	8.7%

**Table 2:** Neuroradiological characteristics and etiology of epilepsy of structural causes

Neuro Imaging features		
Neuroradiological anomalies	72	62.6%
Nature of neuroradiological anomalies		
Cortico-subcortical atrophy	55	47.8%
*Diffuse*	3	2.6%
*Hemispherical*	13	11.3%
*Localized*	39	33.9%
Circumscribed Hypodensity (associated with atrophy)	18	15.6%
Cortico-subcortical calcifications (including 3 associated with atrophy)	5	4.3%
Porencephalic cavity	12	10.3%
Chronic hydrocephalus (associated with atrophy)	4	3.5%
Heterogeneous nodules under ependymal	2	1.7%
Hippocampal sclerosis	3	2.6%
Cortical development malformations	3	2.6%
Brain Tumor	2	1.7%
**Causes**		
Neurocutaneous syndrome (tuberous sclerosis of Bourneville; Sturge Weber syndrome)	2	1.7%
Malformation of cortical development (Polymicrogyry; schizencéphaly)	3	2.6%
Sequelae of cranial and brain trauma	6	5.2%
Sequelae of central nervous system infection	17	14.8%
*Sequelae of meningoencephalitis*	13	11.3%
*Sequelae of neurocysticercosis*	1	0.9%
*Sequelae of cerebral malaria*	3	2.6%
Brain tumors (Ganglioglioma and DNET)	2	1.7%
Sequelae of perinatal cerebral suffering	40	34.8%
Hippocampal sclerosis	2	1.7%

## Discussion

The diagnosed epilepsy etiology varies depending on the geographic locations, the authors, the study methodologies, including whether or not neuro imaging exams are available. In the absence of neuro-imaging examinations in most of the studies in SSA, the certainty of the causative diagnosis of epilepsy is difficult to establish, because generally based on the history of the disease, which is not always recorded and therefore subject to a recall bias [1, 7]. In our study, the etiology of non-genetic epilepsy of the child and adolescent was dominated by the sequelae of antenatal and perinatal cerebral damage and the sequelae of CNS infections. Our results are thus consistent with those of the SSA studies as a whole. In fact, all the descriptive, hospital studies carried out in this region, all methodologies combined, concluded that the etiology or causal factors of non-genetic epilepsy of the child and/or adolescent were dominated by the antenatal or perinatal adverse events, followed by a history of CNS infections and a history of cranial and brain trauma. The proportions of the different causal factors vary according to whether or not genetic epilepsies are included in the study population, depending on whether or not the neuroradiological and biological criteria are used, in addition to the clinical and EEG criteria [1, 4]. According to the series combining clinical and cerebral CT criteria, such as ours, the etiology of non-genetic epilepsy of the child was dominated by abnormalities of pregnancy or antenatal factors and childbirth or perinatal factors and CNS infections [4, 8-11] In two african literature reviews, one in 2006 [1] and the other in 2014, in SSA [7], an implication of perinatal causes was found respectively between 1-36% and between 2%-65% of cases of epilepsy and therefore considered to be major. In our series, the sequelae of perinatal cerebral damage were the leading cause of non-genetic epilepsy of the child and adolescent, or 34.8%, after the undetermined causes, thus confirming the literature data. Hypoxia and hypoglycemia are the frequently cited mechanisms. In the absence of neuro-imaging examinations in several studies, the causal links between a prenatal, perinatal event and epilepsy are difficult to establish. This link is usually based on the history of the disease, which is not always recorded and is subject to a recall bias [12].

Infectious pathology is the other major provider of potentially epileptogenic brain lesions, estimated to be between 1.6 and 6.3% of cases of epilepsy especially in children, in African and Asian work [13]. The history of CNS infection was 20.2% and 11.9% of the causal factors of non-genetic epilepsy in Dakar, respectively in children [8] and in children and adolescents [4] and up to 29.3% of the causative factors of focal genetic and non-genetic focal epilepsies of the child in Cameroon, including non-specific febrile seizures [9]. The sequelae of CNS infections accounted for 14.8% of the causes of non-genetic epilepsy of the child and adolescent in our series. These CNS infections are mainly represented by cerebral malaria (CM), bacterial, parasitic and viral méningoencephalitis [13]. The risk of developing epilepsy in the cohort of survivors of CNS infections is estimated at 26% in developing countries, particularly in SSA [5], compared with 6.8% in developed countries [6]. The risk of developing sequelae with post-bacterial acute meningitis epilepsy is twice as high in Africa (25.1%) and the Southeast Asian regions (21.6%) as in the European regions (9.4%); (p < 0.001) [14]. Nbonda *et al*. [15] found 18% of post bacterial meningitis epilepsy in children previously hospitalized in Yaoundé (Cameroon). In our series, the sequelae of acute meningoencephalitis constituted 11.3% of the causes of non-genetic epilepsy of the child and adolescent, and respectively 4.5% and 9.6% of the causal factors in the series of Ndiaye *et al* [8] and Nguefack *et al* [9]. This could be explained by the persistence of epidemics of meningococcal meningitis, especially in the meningitis belt of La Peyronie, which is particularly serious in children, due to their strong lethality or the high rate of brain damage potentially epileptogenic in survivors. Epilepsy is a recognized neurological sequelae of the CM [16, 17], reported by many analytical hospital studies in children and adolescents, in SSA [18-21]. Our series and that of Ndiaye *et al* in Senegal [8], by identifying the sequelae of CM in 2.6% and about 1% of the causes of non-genetic epilepsy of the child and adolescent, confirmed this observation. In some series, as in ours, the history of cranial and encephalic trauma was the third most important cause of the child's non-genetic epilepsy [8, 10, 12]. Public road accidents, frequent in Africa due to the lack of regulation of traffic, the absence of the wearing of seatbelts or helmets for motorcyclists, are the main cause of head injuries [1]. Cranial and encephalic trauma are relatively common in our patients (5.2% of all patients), especially among adolescents. Indeed, in our context, especially in Burkina Faso, there is a widespread use of bicycles, mopeds and motorcycles in urban centres since adolescence, in contrast to a lack of knowledge of the road code, a lack of wearing of safety helmets, causing public road accidents with severe cranial and encephalic trauma, exposing the subsequent development of post-traumatic epilepsy. In population case-control studies, the antenatal and perinatal adverse events, the history of CNS infections and cranial and encephalic trauma were found to be the main risk factors for epilepsy in children under 18 years of age in SSA [1, 12, 22-26].

of health services, especially in rural areas, often lead to home births without qualified assistance [7]. Certain cerebral structural causes, such as malformations of cortical development, cerebral vascular malformation, neurocutaneous syndromes, brain tumors, hippocampal sclerosis, exclusively identified by MRI, are under diagnosed in SSA, due to low availability and financial inaccessibility of this brain imaging technique [8, 9]. This has been done in our series. Conversely, these causes are reported at fairly high rates in the series using brain MRI [27, 28]. Structural damage such as cortical necrosis with HSV1, infarction with meningitis, hypoxo-ischemic lesions in the CM, and gliosis around neurocysticercosis calcifications can all constitute epileptogenic foci. It is also thought that prolonged proinflammatory stimulation or chronic inflammation, as well as recurrence of the seizures themselves, can lead to a residual pathological condition with rupture of the blood-brain barrier, neuronal death and persistence of neuronal excitability-all of which can contribute to epileptogenesis [29]. Better follow-up of pregnancies, improvement of the conditions of childbirth especially in maternity with assistance by midwiwes or obstetricians, and good coverage in perinatal and post-natal care in equipped paediatric services, would help reduce the burden of epilepsy in SSA. Better prevention of CNS infections in particular an improvement in immunization coverage in quality and quantity that would ensure effective immunization against acute, trial-and-epidemic meningitis, malaria prevention by Effective vector control (impregnated mosquito nets, remediation of the Living Environment,…) and early and effective management of malaria cases before the stage of complications including cerebral malaria, especially in children, would probably contribute to reducing the burden of epilepsy in SSA. Better follow-up of pregnancies, improvement of the conditions of childbirth especially in maternity with assistance by midewives or obstetricians, and good coverage in perinatal and post-natal care in equipped paediatric services, would help to reduce the burden of epilepsy in SSA. Better prevention of CNS infections by effective immunization against endemic and epidemic acute meningitis, malaria prevention through effective vector control (impregnated mosquito nets, remediation of the living environment,…) and early and effective management of malaria cases before the stage of complications including cerebral malaria, especially in children, would also probably contribute to reducing the burden of epilepsy in SSA.

## Conclusion

The causes of non-genetic epilepsies of child and adolescent in Burkina Faso and in SSA are dominated by the sequelae and history of antenatal and perinatal cerebral damages, the sequelae and history of CNS infections and the sequelae of cranial and encephalic trauma. Hospital series combining electroclinical criteria and cerebral CT, such as ours, published in SSA, observed results similar to ours. Similarly in population-based case-control studies in SSA, antenatal adverse events, perinatal events, a history of CNS infections, and cranial and encephalic trauma have been identified as the major risk factors for epilepsy in children under 18 years old. Thus, a better understanding of the acquired etiologies of the child and adolescent would contribute to the formulation of effective preventive maternal and child health policies, and the intensification of the fight against infectious and parasitic diseases, including malaria, especially in chidren, the implementation of which would significantly reduce the burden of this disease. Some highly epileptogenic brain lesions, such as malformations of cortical development, cerebral vascular malformations, neurocutaneous syndromes, certain brain tumors, hippocampal sclerosis, exclusively identified by MRI, frequently reported in developed countries, seem under diagnosed in SSA, because of the low availability and inaccessibility of the MRI. An increased availability and accessibility of MRI would make it possible to know the real place of these structural causes of child and adolescent epilepsies in SSA. *Limits of our study:* our study may not be entirely representative of the epilepsy of the child and adolescent in Ouagadougou, since it did not take into account the epileptic children followed in the primary health centres and the second level medical centres, usually the most numerous. The inclusion criteria, which required the availability of EEG and neuro-imaging (CT scan or MRI) exams for each patient, contributed to the weakness of our sample, or to the failure to take into account certain forms of clinical epilepsy. Some epileptogenic lesions were not visualized due to the very low rate of achievement of brain MRI, yet recognized as a procedure for the neuro-radiological examination of choice in the causal explorations of non-genetic epilepsy. The metabolic and immune causes of epilepsy have not been addressed in our study because of the lack of diagnostic means.

### What is known about this topic

- In SSA, the causal factors of non-genetic epilepsy of the child appear to be dominated by perinatal complications and CNS infections, due to the combination of several factors, such as socio-cultural burdens, the high prevalence of unassisted home births, endemic-epidemic infections, the lack of adequate sanitary infrastructures and qualified human resources;
- In the absence of brain CT scan or RMI in SSA, the causative diagnosis of epilepsy is usually based on the oral history of the disease, therefore subject to a recall bias;
- Some highly epileptogenic brain lesions, such as malformations of cortical development, hippocampal sclerosis,…exclusively identified by MRI, seem under diagnosed in SSA, because of the low availability or inaccessibility of the MRI.

### What this study adds

- Our study confirms that the causes of non-genetic epilepsies of child and adolescent are dominated by the sequelae of antenatal and perinatal cerebral damages, the sequelae of CNS infections and the sequelae of cranial and encephalic trauma;
- Providing data for a better understanding of acquired etiologies of child's epilepsies, it would contribute to preventive maternal and child health policies, and the intensification of the fight against infectious and parasitic diseases, including malaria;
- An increased availability and accessibility of MRI would make it possible to know the real place of other structural causes of child and adolescent epilepsies in SSA.

## Competing interests

The author declare no competing interests.
